# A two-stage simulation analysis of uncertain road damage on the urban emergency delivery network

**DOI:** 10.1371/journal.pone.0267043

**Published:** 2022-05-25

**Authors:** Yinghua Song, Ke Wu, Dan Liu

**Affiliations:** 1 China Research Center for Emergency Management, Wuhan University of Technology, Wuhan, Hubei, People’s Republic of China; 2 School of Safety Science and Emergency Management, Wuhan University of Technology, Wuhan, Hubei, People’s Republic of China; Southwest Jiaotong University, CHINA

## Abstract

When a city encounters a natural disaster, the traffic capacity of the road will change uncertainly over time as the disaster spreads. At this time, it will affect the overall distribution of the urban road network. Therefore, in order to ensure the normal operation of the city, evaluate the objective regularities of impact is of great significance and urgency to emergency decision-makers. The extent and scope of road damaged in the disaster-stricken area varies with time due to the impact of natural calamities. To reveal the regularities impact, this paper provides a two-stage analysis method based on the distribution path of the road network, offering basic data analysis and nonlinear fitting regression analysis on distribution costs, spatial accessibility and distribution efficiency. This study uses the degree of road network damage and the double randomness of road damaged to establish a transportation model for dynamic simulation analysis. The research results show that the delivery regularity of costs, spatial accessibility, and efficiency present the s-curve changes obviously. There are obvious inflection points when the damaged road percentage reaches about 10%-15% and 30%-40%. Therefore, the most suitable delivery route and time can be selected to maximize efficiency and reduce losses.

## 1. Introduction

Natural calamities, such as floods, earthquakes, and debris flows, have occurred more frequently in recent years around the world. These calamities have severely affected normal human live, even caused huge property and life losses. These natural calamities have the massive impact on cities, resulting in many pedestrians and vehicles unable to cross the road, creating a serious threat to public safety. On June 22, 2020, 198 rivers in 16 regions across southern China overflowed beyond the water level warning line, affecting 30.2 million people and causing 141 casualties [1]. On September 13, 2013, a short-duration severe rainstorm hit Shanghai. The rainstorm causing water to accumulate on nearly 100 roads in the city and rendering several routes in the whole road network invalid and paralyzed [2]. In January 2008, a snowstorm struck southern China, which caused many railways, highways, and civil aviation traffic interruptions [3]. On September 23, 2005, Hurricane Rita struck Central and North America. About 24 people lost their lives in the accident, and the hurricane leading to the highway congested [4]. On January 9, 2020, a torrential rain in Angola caused 41 deaths, the destruction of 378 homes, and the flooding of 975 houses [5]. On March 9, 2020, continuous heavy rains in Guaruja and other places of southeastern Brazil caused geological calamities such as mudslides, resulting in 41 deaths, 39 missing and traffic gridlock [6].

In China, the scale of cities has continued to expand in recent years. At the end of 2018, the urbanization rate of permanent residents increased by 8.31% compared with 2011, an average annual increased 1.19%. The urbanization rate of registered population reached 43.37%, increased 3.47% from 2015. An average annual increased 1.16% [4] Due to the large population and high housing density, cities are more susceptible to a series of negative effects brought by natural calamities. When a city suffers major natural calamities, the first task is usually to provide residents with necessary supplies and emergency rescue. However, road conditions may directly limit the efficiency of dealing with these emergencies. Due to the road network may be partially or completely destroyed by natural calamities, it is crucial for the emergency commander’s decision-making that considering the time uncertainty and the location of the natural calamities, estimating the distribution time between the emergency supply point and the demand point.

Furthermore, abundant researches have been studied on the factors that affect the distribution of the transportation road network in recent years. Most of the studies investigated the impact of other factors on the distribution of the road network, such as time windows [5–8], environmental factors [9–12], the vulnerability of the road network [13–15], the reliability of delivery time [16–18], and the psychological impact of the victims [19–21]. Wang et al. pointed out the problem of collaborative multi-depot vehicle routing with time window assignment, and provided a strategy reduced the impact of changing time windows on operating costs effectively [22]. Based on an initial time-dependent vehicle routing model with time windows, Wu et al. synthesized the perishable nature of delivered goods and dynamic travel route choice in urban road network [23]. Rostislav et al. mentioned that transportation road network is particularly vulnerable to a wide range of extreme events (usually natural calamities), and introduced a road network reconstruction based on the ant colony optimization to overcome this issue [24]. Liu et al. compared the dynamic characteristics under the normal traffic condition and the flood-hit traffic condition, to uncover the topology and dynamic evolution of road network vulnerability [25]. Wang et al. explored the impact of traffic accidents, weather as well as flow patterns, and developed a model that can improve delivery efficiency [26]. Kenetsu et al. proposed a simplified model of road network delivery time and reliability analysis by assuming the risk-averse driving factor. Risk-averse drivers choose the route by considering the variance of the route travel time and the average route travel time [27].

On the other hand, the impact of roads on the road network distributions were studied as well, such as road blockage and destruction [28–31], changes in distribution road parameters [32, 33], the road itself changes [34, 35]. Wen et al. investigated the influence of dynamical congestion distribution information based on a set of differential equations, and provided a novel model for common network congestion diffusion that can be relieved by dynamically adjusting congestion distribution information influence [36]. Yakubovich et al. claimed that road network section capacity could be predicted. The capacity of the road network section depends on the technical and operational condition of the road surface -the presence of sinkholes, potholes, ruts, the formation of which depends on the road network sections for natural and climatic conditions [37]. Su et al. designed a method to analyze the disequilibrium of road network to find out the unbalanced or bottleneck points, and the road network structure, traffic flow distribution and the location of sections are considered in the analysis of road network disequilibrium. The disequilibrium is measured by using Gini coefficient and Theil index from road network attributes of influence and capability [28]. Liu et al. proposed a mathematical model, considering vehicle speed, real-time load of vehicle, vehicle travel distance and road slope. The model of the low-carbon time-dependent vehicle routing problem is established with the goal of minimizing the total carbon emissions [38].

Nevertheless, current researches only focused on the degree of damage to the overall road network (the accessibility of the road network at a certain time), or only considered those Blockage and destruction of fixed distribution roads in certain areas within a period time. However, when natural calamities occurred, Road accessibility is constantly changing along with road conditions. Few of the existing studies have focused on combining these two dynamic variables of road network damage and road damage over time. The double-random research method used in this paper combines the damage degree of the road network and the damage degree of the road over time, which solves the above problems well. Under the influence of natural calamities, there is no doubt that the randomness of damage to each road in the overall road network. Although the travel time on certain key routes is difficult to accurately estimate, it is critical to the emergency rescue. Thus, it is urgent to research on which road to choose for delivery and when is the most efficient delivery time when the road network is damaged.

Therefore, in the case of changes in damaged roads, whether there is a regularity in the relevant costs in the process of emergency material distribution is the focus of this paper. In order to explore the impact of damaged roads on the distribution costs, spatial accessibility and distribution efficiency of the distribution regularity, it is important to determined two processes, which are the uncertainty of road damages and selection of the shortest delivery route.

The purpose of this study is to explore the time pattern of rescue routes in the damaged road network. According to the actual road conditions, this study builds a road network, and randomly selects a certain percentage of roads as damaged roads. It calculates the travel time of the selected starting point and destination to summarize time patterns. Through the simulation research of distribution costs, spatial accessibility and distribution efficiency, it is found that three nonlinear fitting regression equation all present obvious S-curve changes, and three obvious inflection points were shown respectively. It shows that the distribution cost, spatial accessibility and distribution efficiency have corresponding regularities in the changing environment of the road network, which is of great help for decision makers to make emergency material distribution decisions when emergencies occur.

## 2. Problem description

In real life, if natural calamities occur, like flood disasters, the roads in the urban will be flooded and damaged due to heavy rain. Moreover, these flooded and damaged roads will not be delivered or the efficiency of traffic will decrease. In addition, the number and extent of damaged roads will change over time and aggravate the disasters.

Such as the rainstorm event in Beijing on July 16, 2018. There were two obvious traffic peak during 8 to 9 am in the morning and 6–7 pm in the noon. The number of congested roads was about twice that of the same on July 9, which was 14%-150% higher than the weekly average level. At this time, based on radar inversion, it could be seen that the rainfall was the highest in these two periods, and the rainfall was less from14 to15 pm [39]. It can be seen that over time, road congestion caused by road damage due to heavy rain is positively correlated with rainfall.

Delivery roads will inevitably change due to the increase in the number of damaged roads, which will correspondingly affect the cost and efficiency of supply allocation when disasters occur. If the regularity of cost and efficiency can be found in the delivery of materials when the number of damaged roads increases, then the time pattern of rescue routes can be better explored, and transportation time can be estimated to help emergency commanders make decisions.

### 2.1 The construction of transportation networks

The construction of road network is the foundation for estimating the travel time. It is generally accepted that any road network constructed according to actual urban road conditions is constructed at the initial stage, each node represents an intersection, and each link represents a road segment.

The attributes of each node include node ID, longitude and latitude. The attributes of each link include link ID, start node ID, end node ID, distance, speed level, and passable state.

**Fig 1:**
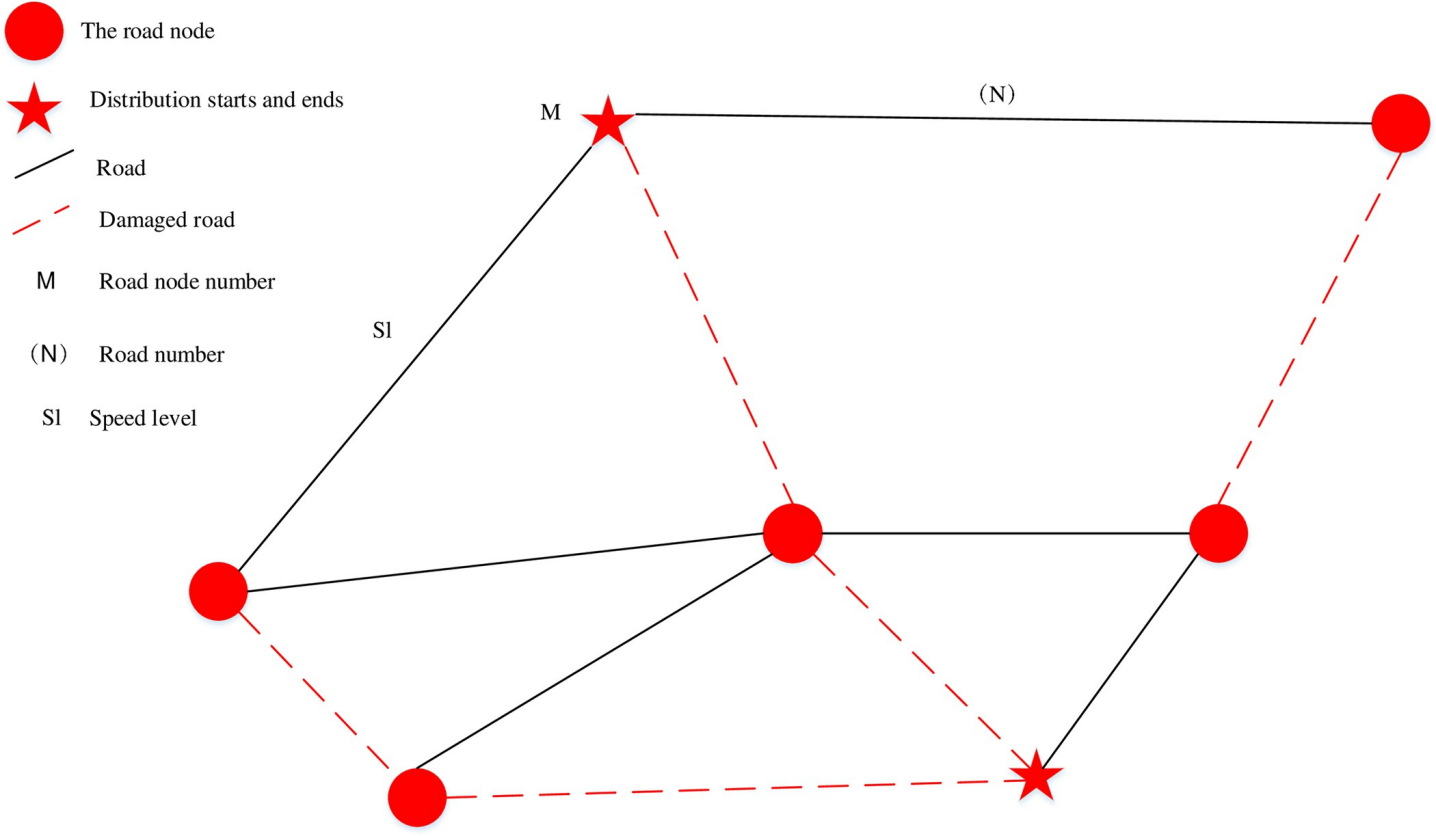
Distribution diagram.

The distribution diagram is shown in Fig 1.

The time and location of large-scale natural calamities are usually unpredictable. As mentioned above ‘any road network generated road network’, the travel route and time used for rescue and resource allocation can be analyzed.

### 2.2 Symbols and variables

In order to facilitate the follow-up study and make it easier for readers to understand, all symbols and symbol explanations are now placed as following.

N_d_: Number of damaged roads;

N_n_: Total number of roads in the road network;

P: Percentage of road damage (%);

map[i,j]: Shortest distance from i to j;

K: Breakpoint of exhaustive i,j;

P_r_: Percentage of delivery availability (%);

N_r_: Number of delivery availability;

N: Number of experiment;

D_0:_ Original distance (m);

D_n_: Updated distance after considering the driving speed (m);

C: Driving speed of the road (km/h)

### 2.3 Uncertainty of damaged roads

Any percentage of the roads in the random road network constructed above are regarded as impassable damaged roads. In order to reflect the uncertainty of road damages, with the passage of time, the degree of road damage gradually increased, and these damaged roads are randomly selected. In this study, 5% to 50% of the road network was randomly selected as damaged roads, and every 5% was used as an interval.

The fixed road damage session and the percentage of damaged roads are used as variables, and then the corresponding number of damaged roads is calculated. The calculation method of the number of damaged roads shown by (1) and the results are shown in Table 1 below:

**Table 1:** Number of damaged roads.

P (%)	5	10	15	…	45	50
**N**_**d**_	N_n_·5%	N_n_·10%	N_n_·15%	…	N_n_·45%	N_n_·50%


$$N_{d}=N_{n}\cdot P$$ (1)

Where N_d_ is the Number of damaged roads. N_n_ is the total number of roads in the road network. P is the percentage of road damage (%).

### 2.4 Essential questions

#### 1) Distribution costs

Based on a general road network shown in Fig 1, this test randomly selected two nodes and calculate the shortest delivery road between these two nodes. The Floyd Shortest Path Algorithm is able to find the shortest delivery road.

The current damage time is fixed. In the disaster events, the proportion of damaged roads in the current road network increases from 5% to 50%. Randomly select damaged roads to all roads for each variable.

Then, this test uses Floyd-Warshall algorithm edited in C++ to calculate the shortest distance between two nodes as the distribution costs. By changing different road conditions and adopting series of experiments, it can be acquired multiple component datas, using Matalab software to process and sort the data.

#### 2) Spatial accessibility

In each simulation, due to different road damage percentages, there will be impassability between the two nodes, which will affect the proportion of transportation routes that can be passed during the delivery process. The proportion of unreachable routes in each percentage is calculated, where nonlinear fitting regression analysis is performed.

#### 3) Distribution efficiency

All roads are randomly selected, and each road is divided into four levels randomly according to national road regulations. These roads are divided into four levels according to different speeds from 0 to 80 km per hour, namely 60-80km/h, 40-60km/h, 30-40km/h, and 0-30km/h. For the convenience of subsequent experimental calculations, if the first-level speed is 1, the second, third, and fourth-level speeds can be approximately 0.8, 0.6, and 0.4 times of the first-level speed respectively.

In order to reflect different delivery speeds on roads, the reduction of the traffic speed can be converted into the growth of the road length. Therefore, according to the first-level, speed is 1, the second, third, and fourth-level speeds are 0.8, 0.6, and 0.4 times of the first-level speed respectively. The length of the first level was unchanging. The road length of second-level is 5/4 of the length in first-level. The road length of third-level is 5/3 of the first-level. The road length of fourth-level is 5/2 of the first-level. The details are shown in Table 2 below.

**Table 2:** Data of update distance.

Road grades	V_1_	V_2_	V_3_	V_4_
**C(km/h)**	60–80	40–60	30–40	0–30
**D**_**n**_	D_0_	D_0_·5/4	D_0_·5/3	D_0_·5/2

By converting the delivery speed into the length of delivery roads as described above, a new road network with a different length from the previous road network can be obtained.

### 3. Dynamic transportation change model of urban emergency logistics

#### 3.1 Technical flowchart

This report provides a two-stage analysis method based on the distribution path of the road network, and considering the double randomness changes of spatial and temporal dynamic. A large number of experiments employed and basic data analysis provided. on linear programming analysis on transportation costs, accessibility and efficiency. And then come to the conclusion of regularity.

**Fig 2:**
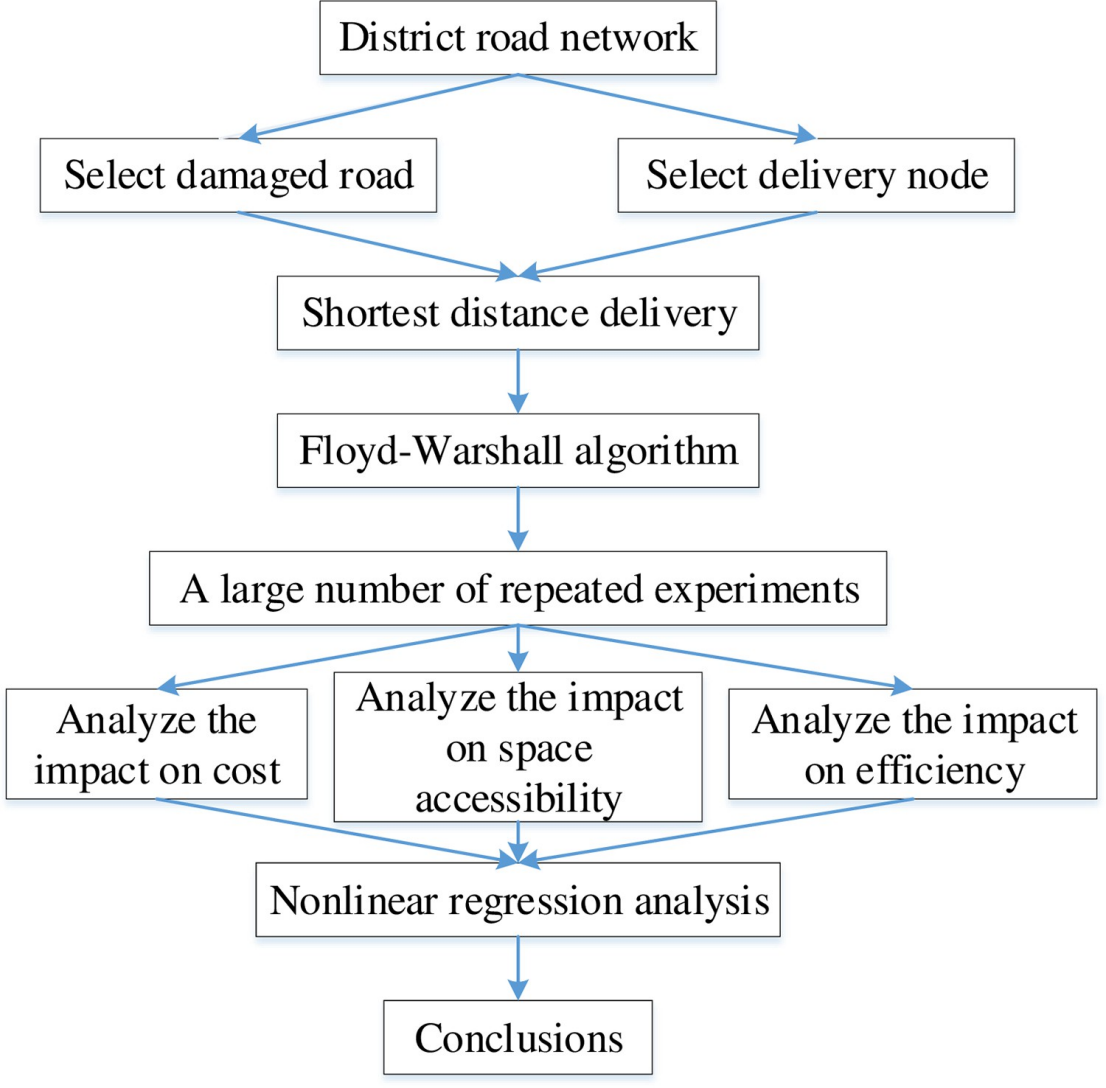
Technical flowchart.

The technical flow chart is shown in Fig 2 below.

### 3.2 Random simulation tests

The first step focuses on constructing a random road network, the second step is to determine the proportion of randomly damaged roads, and the third step is to randomly select two points in the network as the start and end points of the delivery. It is impassable due to different proportions of road damage for the road network, several simulation experiments are carried out respectively. This research uses the Floyd-Warshall algorithm to find the shortest path, as well as analyzes transportation cost, accessibility and efficiency. It also identifies the delivery regularity of these three through nonlinear fitting regression analysis. In the last step, the nonlinear fitting regression equation of the experimental results is analyzed in detail to obtain the delivery regularity.

Two remote nodes are randomly selected as starting and ending points of supply deliveries. When the degree of road damage is different, the delivery time of different supplies between the two nodes with the shortest distance is calculated, including the distribution costs, space accessibility, and distribution efficiency.

This study performed at least 30 random simulations for each percentages. To ensure the accuracy of the test, as the percentage of damaged road increases, the number of experiments should be appropriately increased. Each simulation calculates the cost based on the shortest distance between the assigned roads, and then analyze the delivery of transportation costs, accessibility and efficiency. The details are shown in Table 3 below.

**Table 3:** Number of simulation tests.

Percentage of damaged roads (%)	Number of experiments
5	30
10	35
15	40
…	…
45	70
50	75

### 3.3 Problem solving

#### 1) Analysis of distribution costs

Floyd-Warshall algorithm shown by (2) below.


map[i,j]≔min{map[i,k]+map[k,j],map[i,j]} (2)

Where map[i,j] is the shortest distance from i to j. K is the breakpoint of exhaustive i, j. The initial value of map[m,n] should be 0.

The data results are displayed in Origin, take the average shortest distance between the experimental results from each group percentage before performing nonlinear fitting regression analysis.

By comparing a large number of nonlinear fitting regression equations, it was found that the nonlinear fitting regression curve of a certain S-shaped curve was very consistent with the experimental results. The nonlinear fitting regression equation of distribution costs and efficiency is demonstrated in (3).


$$y=A_{2}+\left(A_{1}-A_{2}\right)/(1+exp\left(\left(x-x_{0}\right)/d_{x}\right)$$ (3)

#### 2) Analysis of space accessibility

The Analytical method shown by (4) below:

$$P_{r}=\frac{N_{r}}{N}\times 100\%$$ (4)

Where P_r_ is the percentage of delivery availability (%), N_r_ is the number of delivery availability. N is the number of experiment.

The experimental results were put into Origin for analysis. By comparing a large number of nonlinear fitting regression equations, it was found that the nonlinear fitting regression curve of a certain S-shaped curve was very consistent with the experimental results. The nonlinear fitting regression equation of spatial accessibility is (5).


y=A2+(A1−A2)/(1+((x/x0)^p) (5)

#### 3) Analysis of efficiency

The calculation method of the Data of update distance shown by (6):

$$D_{n}=C\cdot D_{0}$$ (6)

Where D_0_ is the original distance (m); D_n_ is the updated distance after considering the driving speed (m). C is the driving speed of the road (km/h).

Based on this method, simulation experiments are conducted repeatedly. Same as the method used to calculate the distribution costs, this stage uses the Floyd-Warshall algorithm to calculate the shortest distance between two nodes as the distribution efficiency. The data results are displayed in Origin, and the programming is performed.

The nonlinear fitting regression equation is same as (3).

## 4. Situational response analysis

### 4.1 Scenario building

From 8:00 to 17:00 on July 20, 2021, a heavy rainstorm occurred in Zhengzhou City, Henan Province, China, with a localized heavy rainstorm. From 16:00 to 17:00 on July 20, 2021, the rainfall in Zhengzhou reached 201.9mm in one hour. Heavy rain caused extensive traffic suspension, and most of the roads and subways were blocked. The disaster had a huge impact on local residents’ normal lives. At present, there is a batch of emergency supplies that need to be sent to schools and communities in the area to ensure the normal life of the people. The flood disaster scenarios caused by sudden heavy rain are shown in Table 4 below.

**Table 4:** Summary table of epidemic scenarios.

Items	Contents
**Occurrence time**	July 20, 2021
**Scene of action**	Jinshui District, Zhengzhou City, Henan Province, China
**Event occurrence**	Emergency supplies were timely supplied across the region
**Requirement type**	Life jackets, food and other emergency supplies
**Requirement number**	A number of emergency supplies
**Degree of urgency**	Urgent (To be delivered as soon as possible)
**Consequences**	Sent to various material demand points, otherwise it will seriously affect people’s lives and health
**Missions**	Deliver emergency supplies to each disaster-stricken material demand points as soon as possible in a safe and orderly manner

**Fig 3:**
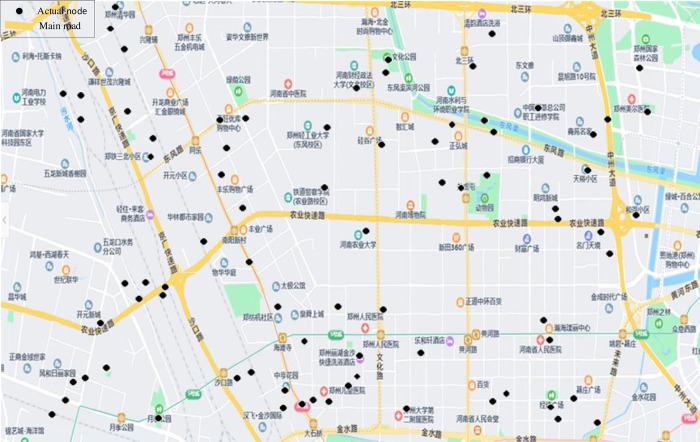
Actual node distribution.

**Fig 4:**
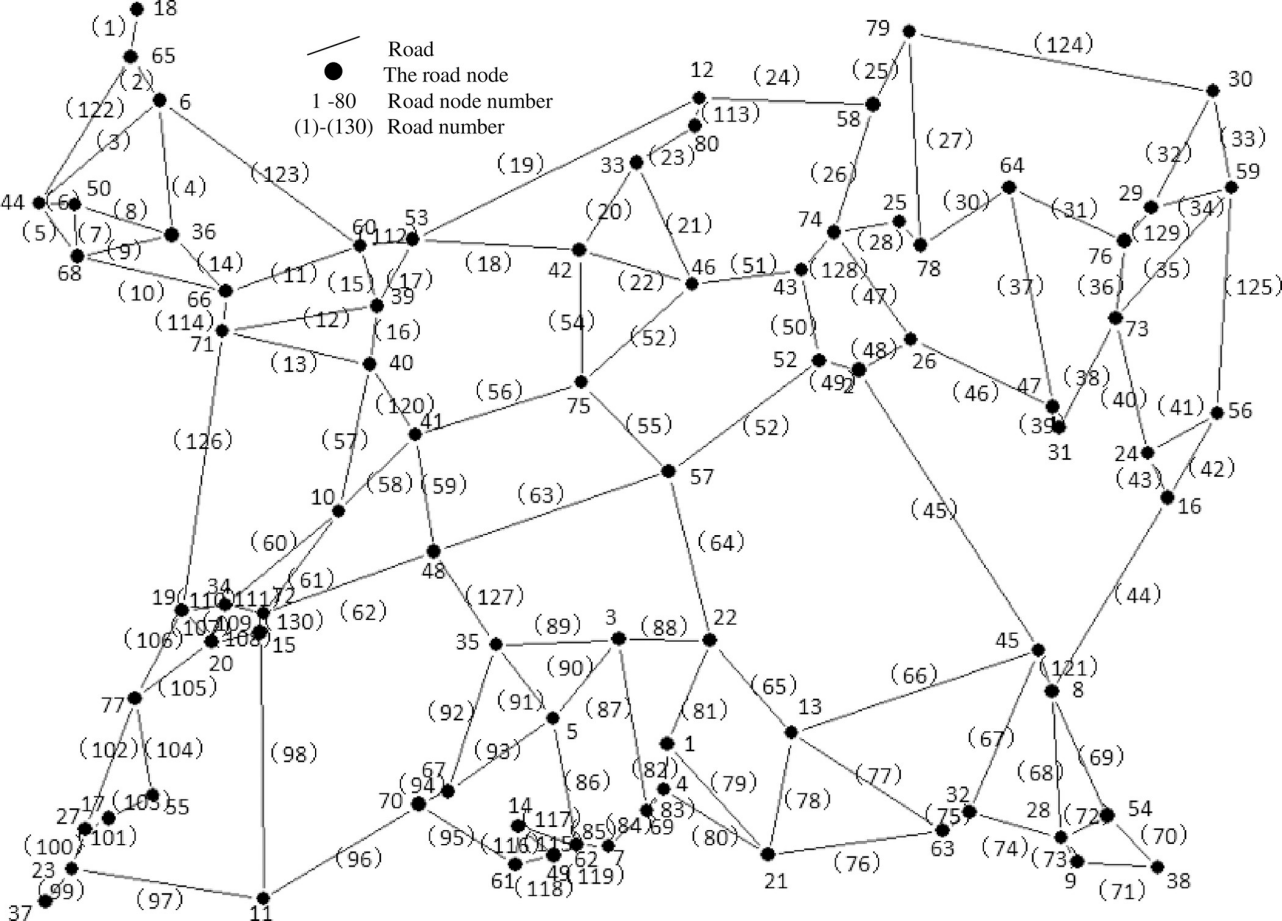
Topology diagram of road network.

Jinshui District, Zhengzhou City, Henan Province, China was selected as the research object. Select 80 schools, communities and business districts in the area as the research objects. These locations are used as distribution nodes, and emergency supplies are distributed through these distribution nodes. Details are shown in Fig 3. Due to the short distance between nodes, in order to facilitate calculation, the traffic road between nodes is simplified into a distribution straight line distribution road for research. Then, a topology diagram is established based on the actual road network information, as shown in Fig 4.

The road network consists of 80 nodes and 130 roads. Figs 3 and 4 display the sketch map. The road topology in Fig 4 is constructed by the actual location nodes in Fig 3. The selected study area is located in Jinshui District, Zhengzhou city, centered on Henan Agricultural University. It is 10 kilometers long, 5 kilometers wide and covers a total area of 50 square kilometers. The link attributes of nodes and distribution roads are shown in Table 5 (see Appendix A for full raw data in S1 Appendix). The speed level represents the speed limits of each road segment. The passable state includes passable (value 1) and impassable (value 0). Normal roads are passable while roads damaged by natural calamities are impassable.

**Table 5:** Attributes of links of the road network.

Attribute	Road number	Start node number	End node number	Distance(m)	Speed level(km/h)
**Value**	1	18	65	527	60–80
	2	65	6	536	60–80
	3	44	6	1504	60–80
	…	…	…	…	…
	129	76	29	434	30–40
	130	72	15	216	60–80

### 4.2 Delivery regularity of costs

In the road network of Fig 4, randomly selected node 65 and node 38 as two distant nodes. The Floyd-Warshall algorithm is used to calculate the shortest delivery road between these two nodes, which is 14978 meters. When the current damage time is fixed, the proportions of damaged roads (variables) are 5%, 10%, 15%, 20%, 25%, 30%, 35%, 40%, 45%, and 50%. To reflect the uncertainty of damaged roads, the number of damaged roads is randomly selected under each variable.

When the proportion of damaged roads increases from 5% to 50%, the number of damaged roads is correspondingly 6 to 65. A total of 30 to 75 random simulations were performed at this stage, and then the average shortest distance under each percentage was calculated. Finally a nonlinear fitting regression analysis was performed on the average shortest delivery distance under each group of variables.

Simulation test and test data of distribution cost of 65–38 are shown in Tables 6 and 7 below:

**Table 6:** Simulation tests of distribution cost of 65–38.

Percentage of road damage (%)	Number of damaged roads	Number of experiment
5	6	30
10	13	35
15	19	40
20	26	45
25	32	50
30	39	55
35	45	60
40	51	65
45	58	70
50	65	75

**Table 7:** Test data of distribution cost of 65–38.

Percentage of road damage (%)	Data of shortest distance of 65–38 from the current damaged road percentage value(m)	Average delivery distance (m)
5	14978,14978,16108,16108,16097,16168,14978,16097,16108,16129,17958,14978,14978,15166,14978,14978,14978,14978,14978,16108,16149,14978,16097,16097,16108,17282,18166,14978,14978,14978	15721.27
10	16168,14978,15656,16129,16807,17227,16097,22354,14978,16108,18995,15166,17505,15656,15935,16097,18148,15935,15935,17355,16097,14978,16014,16956,18546,17282,15656,15935,14978,14978,16168,16846,17555,17125	16539.5
15	17106,15826,18148,26044,17065,16294,17633,17926,18462,16108,15166,15844,17298,24692,17185,15826,15826,14978,14978,16149,16285,17764,17784,16956,17830,18999,17070,20812,17065	17555.83
20	15935,15166,20199,18125,20845,18849,19056,19444,18819,21303,16285,19573,20993,18417,16294,20955,16640,20249,16149,14978	18309.77
	21422,16538,18481,19095,17282,17268,16786,19751,18246	
25	20013,16945,22283,21270,25733,17133,21874,18246,18146,19999,18381,16294,17549,16108,16097,16041,16807,19580,21796,20445,21818,16149,20550,18386,24049,19500,17177,19144	19196.89
30	25196,18848,17657,17830,23197,20178,21837,24650,19751,19161,19600,15656	20296.75
35	22300,20231,18272,18615,19421,20550,19326,19553,18403,19163	19583.4
40	20931,18561	19746
45	None	None
50	None	None

**Fig 5:**
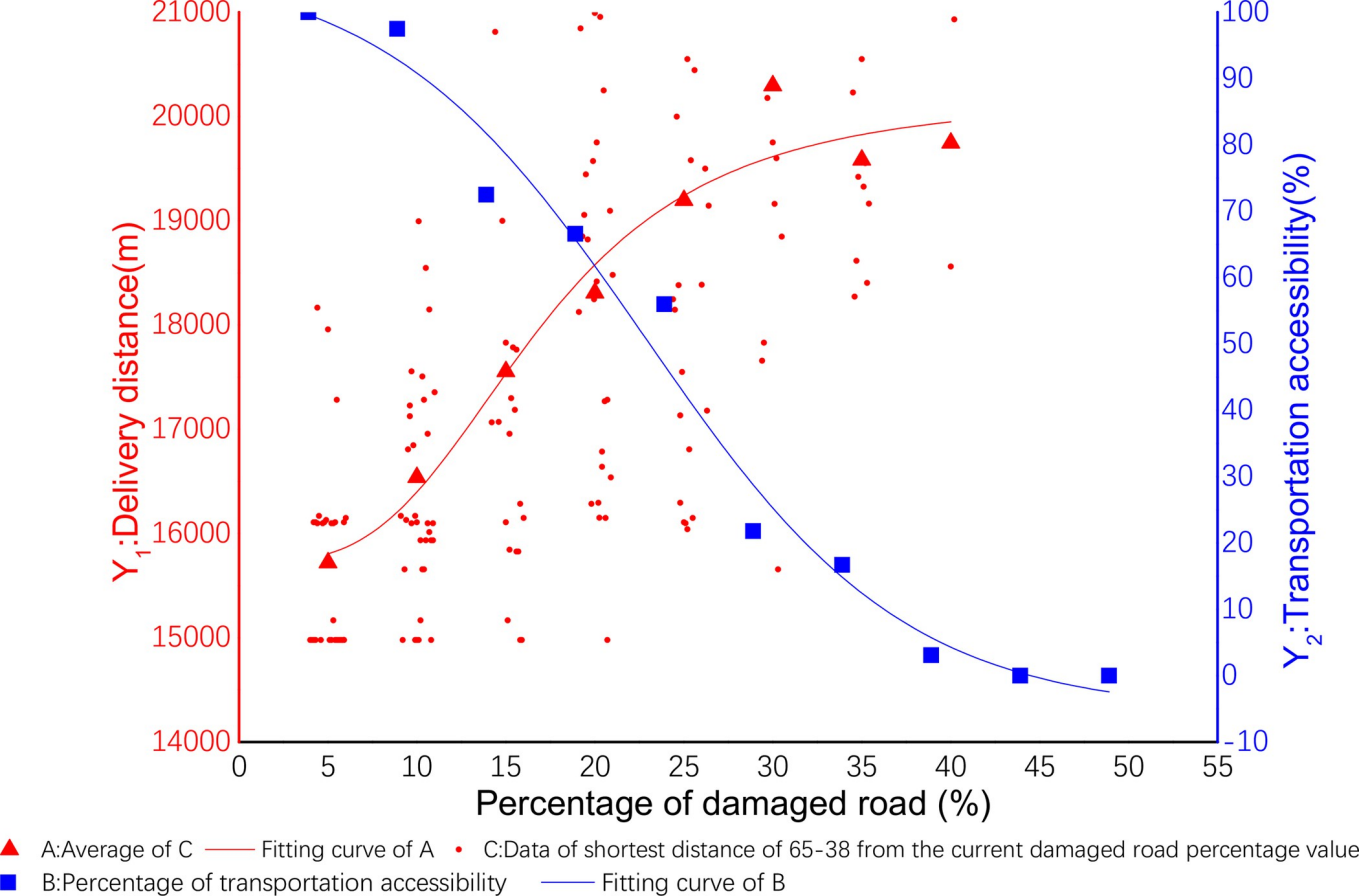
Delivery cost and spatial accessibility of 65–38.

The data results are displayed in Origin (shown in Fig 5 below). The abscissa X is the proportion of damaged roads, the ordinate Y_1_ is the shortest delivery distance, and the data of small red dot is all the shortest delivery distances in the percentage interval of damaged roads. The data of red triangle is the average value of all the small red points in the percentage interval of the damaged road, then a nonlinear regression is performed on all the data of red triangles to obtain the red curve. And the blue curve is presented in section 4.3 below.

The coefficients of nonlinear fitting regression equation of distribution cost are shown in Table 8 below. The nonlinear fitting regression equation corresponding to this coefficient shown by (3). Among them A_1_ = 107.60465, A_2_ = -5.57967, X_0_ = 23.91282, d_x_ = 4.32567. See (7) for details:

**Table 8:** Fitting equation parameters of distribution cost of 65–38.

A_1_	A_2_	X_0_	d_x_
107.60465	-5.57967	23.91282	4.32567


y=‐5.57967+113.18432/(1+exp((x−23.91282)/4.32567) (7)

In the selected road network, the analysis of the test results found that the number of damaged roads has a great impact on the distribution costs of the delivery route. Nonlinear simulation regression analysis found that the regression curve of transportation cost data is S-shaped. Among them, distribution costs increase with the increase of damaged roads. When the proportion of damaged roads raises from 5% to 10%, the distribution costs will increase slowly; when the proportion of damaged roads increases from 10% to 25%, the transportation cost will increase rapidly; when the proportion of damaged roads increases from 25% When it increases to 40%, transportation costs increase slowly and steadily; when the proportion of damaged roads is greater than 40%, there is no passable road. The results are displayed in the Table 9 below.

**Table 9:** S-curve change trend of distribution cost of 65–38.

Percentage of road damage (%)	The trend of accessibility
5–10	Increase slowly
10–25	Increase rapidly
25–40	Increase slowly; stable
40-	None

According to test results, when the road network environment changes due to calamities, the number of damaged roads gradually increases over time. Taking into account the distribution costs, the percentage of road damage during the transportation of urban emergency supplies should be controlled within 10% of the time and distributed as soon as possible. Distribution costs below this percentage are low, and the increase in distribution costs is small. However, when the percentage of road damage exceeds 10%, the distribution costs will increase rapidly, which means that the distribution costs in the range of 10% to 25% is the fastest growing. When the percentage of road damage is between 25% and 40%, the distribution cost increases slowly and reaches a peak. When this percentage is higher than 40%, the road network is severely damaged, resulting the delivery will not be completed. Therefore, transport vehicles should be delivered as soon as possible before the proportion of road damage reaches 40%. It is best to deliver when the distribution costs is the lowest (the proportion of road damage is less than 10%). These test results can better help emergency commanders make decisions properly to control the distribution costs.

### 4.3 Delivery regularity of spatial accessibility

In the test of distribution costs, it is found that there are unreachable roads under different road damage percentages in the delivery process from node 65 to node 38, which will affect the spatial accessibility of entire road network.

This stage analyzed the proportion of transport routes that are not reachable under each percentage test. The delivery is shown in Fig 5. It shows that the abscissa X is the proportion of the damaged road, and the ordinate Y_2_ is the accessibility. The data of blue square is the distribution accessibility value for every 5% when the percentage of damaged roads in the experiment is from 5% to 50%. The specific values are shown in the Table 10 below. And then a nonlinear simulation regression is performed on all the data of blue squares to obtain the blue curve.

**Table 10:** Simulation tests of spatial accessibility of 65–38.

**Percentage of road damage (%)**	5	10	15	20	25	30	35	40	45	50
**Number of experiment**	30	35	40	45	50	55	60	65	70	75
**Number of delivery availability**	30	34	29	30	28	12	10	2	0	0
**Accessibility (%)**	100	97.14	72.5	66.66	56	21.82	16.67	3.08	0	0

The coefficients of nonlinear fitting regression equation of spatial accessibility are shown in Table 11 below. The nonlinear fitting regression equation corresponding to this coefficient shown by (5). Among them A_1_ = 15727.32516, A_2_ = 20187.28722, X_0_ = 16.83692, p = 3.32054. See (8) for details:

**Table 11:** Fitting equation parameters of spatial accessibility of 65–38.

A_1_	A_2_	X_0_	p
15727.32516	20187.28722	16.83692	3.32054


y=20187.28722+(−4459.96206/(1+((x/16.83692)^3.32054)) (8)

Known from experiments, as the number of road damages increased, the proportion of opportunities to reach of successful delivery decreased accordingly. When the percentage of damaged roads increases from 5% to 15%, the percentage of opportunities to reach of successful delivery decreases slowly; when the percentage of damaged roads increases from 15% to 35%, the percentage of opportunities to reach of successful delivery decreases rapidly; When the percentage of damaged roads increased from 35% to 45%, the percentage of opportunities to reach of successful delivery slowly decreased and remained stable. When this percentage is greater than 45%, the percentage of opportunities to reach of successful delivery is close to zero. The details are shown in the following Table 12.

**Table 12:** S-curve change trend of spatial accessibility of 65–38.

Percentage of road damage (%)	The trend of accessibility
5–15	Decrease slowly
15–35	Decrease rapidly
35–45	Decrease slowly; stable
45-	None

The results of this stage show that when a disaster occurs in an urban road network, the number of damaged roads will gradually increase over time, which leads to a gradual decline in the opportunities to reach of successful delivery Taking into account the effect of space accessibility the percentage of road damage should be below 15% during the delivery of urban emergency supplies. At this percentage, the accessibility of delivery is higher, and the decline in opportunities to reach of successful delivery is slower. When the percentage of road damage exceeds 15%, opportunities to reach of successful delivery drops rapidly. When this percentage is higher than 45%, the road network will be inaccessible, and the delivery of emergency supplies will not be completed. Therefore, when a disaster occurs and the road network is damaged, the spatial accessibility before the percentage of road damage reaches 15% is the best. Higher than 15% will lead to a sharp decline in the spatial accessibility of the road network, further leading to delays in delivery or other problems. After the proportion of road damage is higher than 45%, road transportation will not be implemented. It can be seen that the delivery should be executed as soon as possible before the proportion of road damage reaches 45%. These results are helpful for decision-making and can ensure the best opportunities to delivery.

### 4.4 Delivery regularity of efficiency

Considering the road grades of each road, the length of each road in the update road networks is shown in Tables 6–13 below (see Appendix B for full raw data in S1 Appendix).

**Table 13:** Data of road network.

Road number	Road grades	D_0_(m)	D_n_(m)
1	1	527	527
2	1	536	536
3	1	1504	1504
…	…	…	…
129	3	434	723
130	1	216	216

Update the road network according to the data in Table 13 to obtain a new road network. In the new road network, 5% to 50% roads are randomly selected as damaged roads, and 30 to 75 random simulation experiments are conducted under each percentage of damaged roads, in which node 65 and 38 are allocated for delivery test. Floyd-Warshall algorithm is utilized to calculate the shortest delivery distance, then use nonlinear fitting to analyze distribution efficiency. The experiment data are shown in the following Table 14 below.

**Table 14:** Test data of distribution efficiency of 65–38.

Percentage of road damage (%)	Data of shortest distance of 65–38 from the current damaged road percentage value(m)	Average delivery distance (m)
5	21846,21846,23866,23928,23705,22639,21846,23705,23822,24730,25301,23705,21846,24678,22639,21846,21846,21846,21846,23822,22639,21846,26701,23705,23822,24518,26094,21846,21846,21846	23205.7
10	22639,21846,23678,24730,27920,25681,23705,26094,24518,23822,26553,22343,25301,30476,22389,23705,25301,23705,22389,26738,23705,21846,24175,25654,25407,24518,23678,22389,21846,21846,22639,24471,24518,23182	24217.85
15	24079,23678,34324,39936,24365,23536,26917,28477,28381,23928,22343,25066,24615,30488,24968,25654,23678,23705,21846,22639,24202,27161,25658,25654,26094,27756,25407,31951,24365	26236.9
20	22389,22343,29767,26505,32370,26200,29621,27919,29603,32127,24202,29542,32150,26474,24181,33867,25301,31310,23944,21846,32111,23928,27885,28306,25786,24498,25654,23866,28692,25787	27272.47
25	29430,25537,25301,28731,26034,32979,25787,26447,30476,23536,27954,23822,23705,24175,25786,28026,36818,31844,33122,23944,29727,27742,39678,32335,28633,26611	28391.5
30	41629,29357,15915,26094,34417,29265,34914,35471,28713,28871,29045,24569	29855
35	35810,32923,27989,30485,29868,29727,27304,31468,29135,28239	30294.8
40	27920,31490,31490	30300
45	None	None
50	None	None

**Fig 6:**
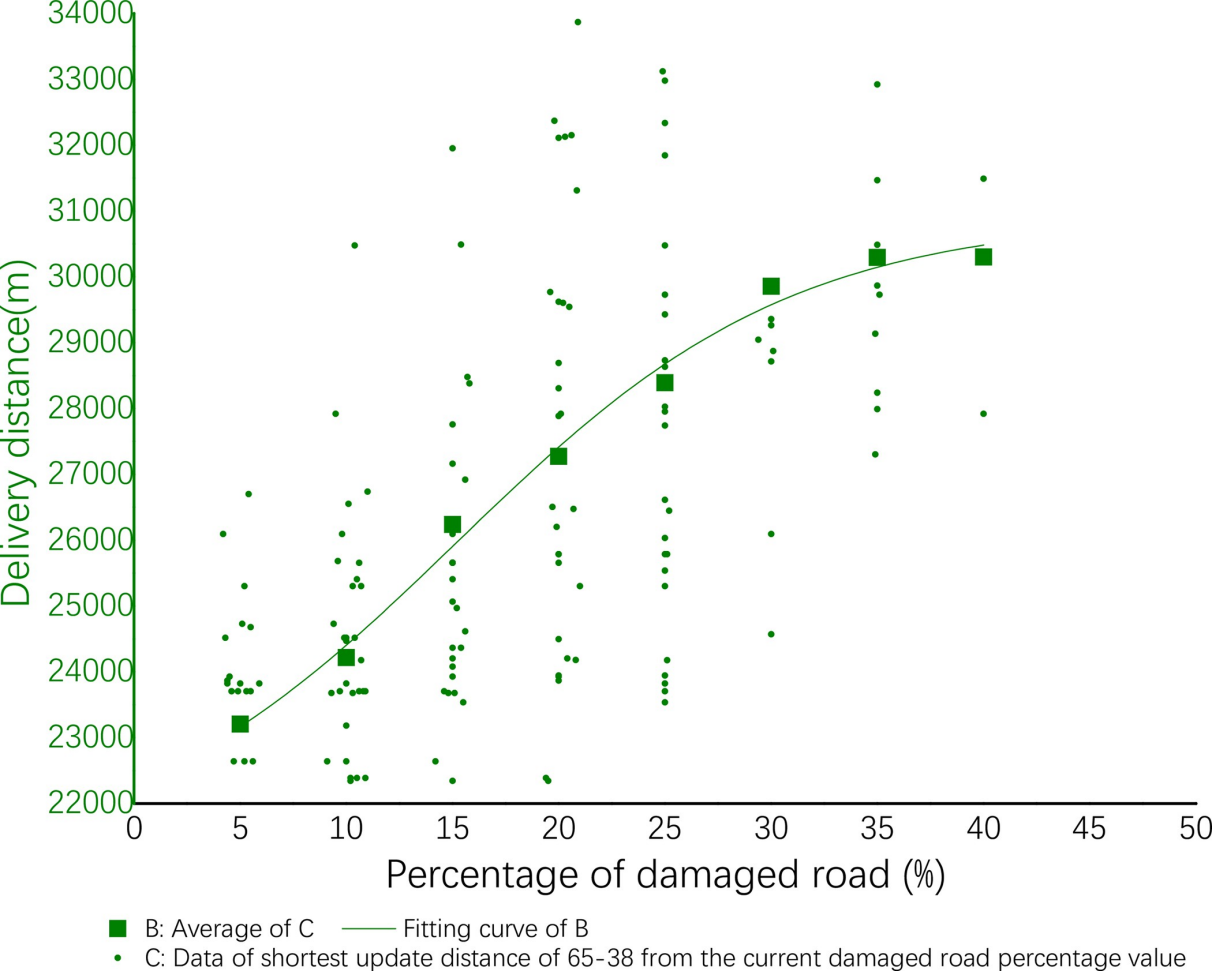
Delivery efficiency of 65–38.

The results are displayed in Origin and displayed in the Fig 6 below. In the Fig 6, the horizontal axis X is the proportion of damaged roads, the vertical axis Y is the shortest delivery distance, and the data of small green dots are all the shortest delivery distances based on different percentages of the damaged road.

The data of green squares are average of all the data of small green dots that account for the percentage of damaged roads. In this stage, a nonlinear regression is performed on all data of green squares to obtain a green curve.

The coefficients of nonlinear fitting regression equation of efficiency are shown in Table 15 below. The nonlinear fitting regression equation corresponding to this coefficient shown by (3). Among them A_1_ = 21016.52756, A_2_ = 30896.89536, X_0_ = 15.16212, d_x_ = 7.94418. See (9) for details:

**Table 15:** Fitting equation parameters of efficiency of 65–38.

A_1_	A_2_	x_0_	d_x_
21016.52756	30896.89536	15.16212	7.94418


y=30896.89536+(−9880.3678/(1+exp((x−15.16212)/7.94418)) (9)

From the experimental results, it can be found that distribution efficiency is reduced due to the increase of the damaged road rate. When the percentage of damaged roads increases from 5% to 15%, the distribution efficiency will slowly decrease; when it increases from 15% to 30%, the distribution efficiency decreases rapidly; when it raises from 30% to 40%, the distribution efficiency slowly decreases and then stabilizes; when it is higher than 40%, there is no passable road. The details are shown in the following Table 16.

**Table 16:** S-curve change trend of accessibility of 65–38.

Percentage of road damage (%)	The trend of efficiency
5–15	Decrease slowly
15–35	Decrease rapidly
35–45	Decrease slowly; stable
40-	None

Based on the distribution cost test, the delivery efficiency of the entire road network can be obtained by attaching a random driving speed limit to each road to ensure it closer to a real road network. The regularity of delivery efficiency is roughly the same as the regularity of delivery costs. However, after considering the delivery speed of each road, the delivery trend will vary depending on the percentage of damaged roads. In the process of urban emergency material delivery, if the delivery efficiency is taken into account, the percentage of road damage should be within 15% of the road network. Below this percentage, the delivery efficiency is very high, and its decline rate is very slow.

When the percentage of road damage exceeds 15%, the distribution efficiency will drop rapidly. The distribution efficiency is stable until this percentage reaches 30%. After 40%, the road network is inefficient, and the delivery of emergency supplies cannot be completed. Therefore, when a disaster occurs, the delivery efficiency is the best when the road damage percentage is below 15%. When it is higher than 15%, the efficiency of the road network drops sharply, which may lead to increased delivery costs and time, as well as other delivery problems due to inefficiency. When it is higher than 40%, the delivery cannot be completed due to impassable roads. It can be found that material delivery should be completed before the road damage rate reaches 40%. This experiment can better understand the efficiency delivery of the entire delivery network, and provide a helpful suggestion to facilitate delivery efficiency for decision makers.

### 4.5 Control experiment

In this stage, the other two further nodes are selected for control the experiment. Firstly, selecting damaged roads randomly with the same percentage, then setting up the same number of tests and implement them repeatedly. Keep the road network of the previous experiment unchanged, the delivery speed unchanged, the updated delivery distance unchanged and all other assumptions remain unchanged, by changing different delivery starting points and ending points to carry out the same experiment, in order to obtain similar delivery rules. Finally, the impact of the damaged roads number on distribution costs, accessibility and delivery route efficiency is analyzed. The same regression analysis is performed according to the tests mentioned above, the following results are obtained.

In order to control the test, two far away points are randomly picked. The node 16 to node 44, and node 30 to node 23 are selected. The same percentage of damaged roads are randomly selected, and the same number of experiments are set and implemented. Finally, the control test analyzes the impact of the number of damaged roads on delivery costs, accessibility and efficiency.

**Fig 7:**
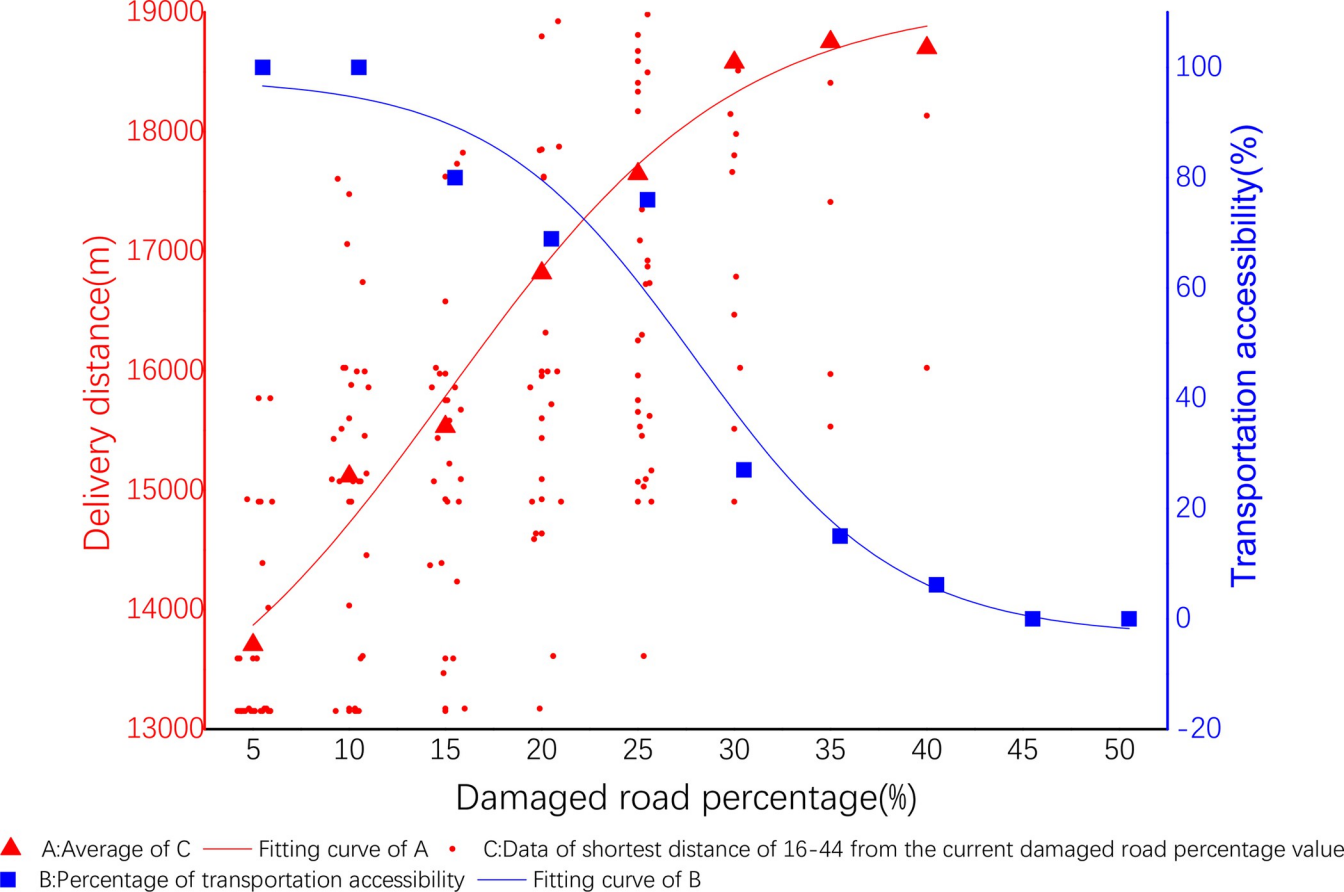
Delivery cost and spatial accessibility of 16–44.

**Fig 8:**
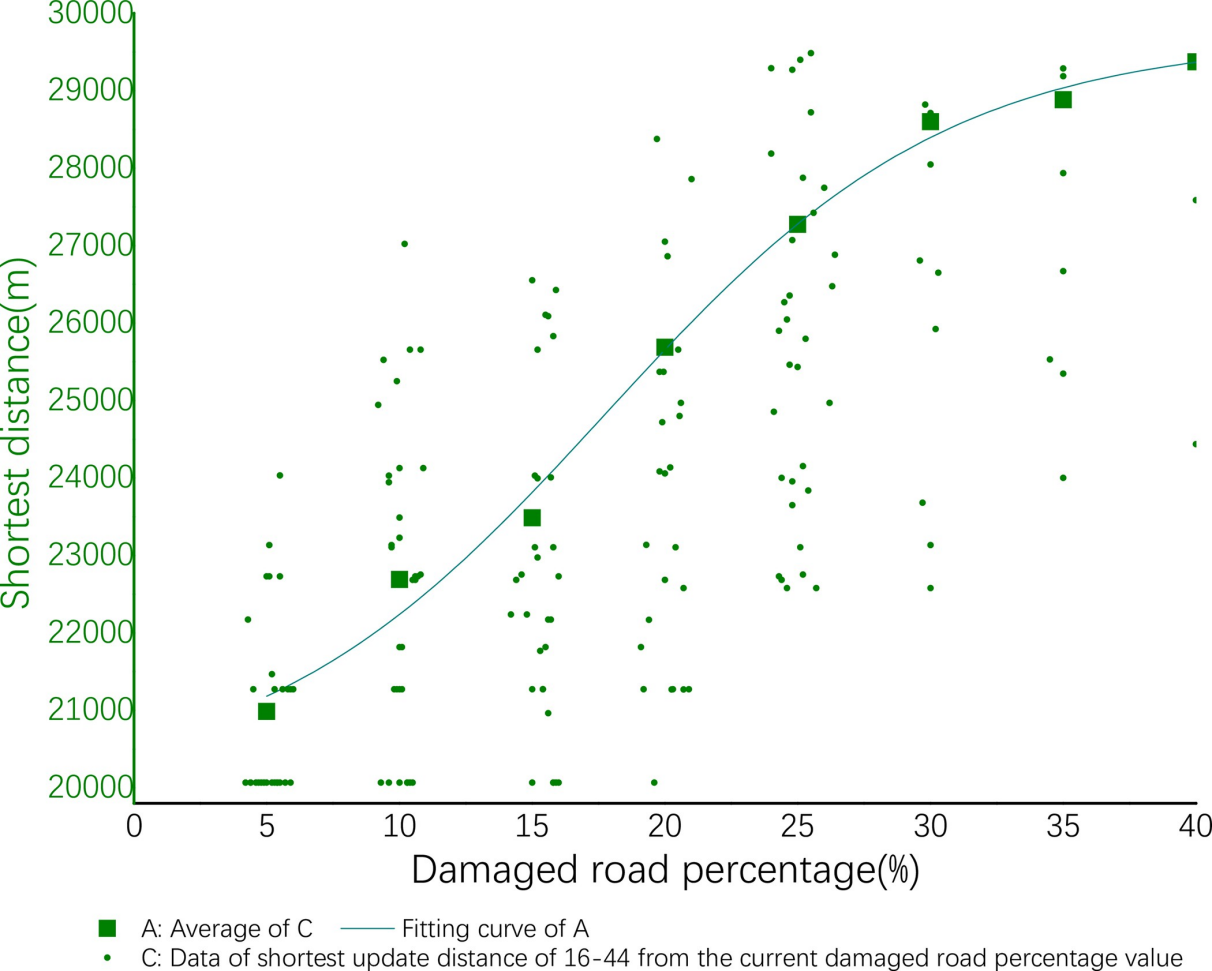
Delivery of efficiency of 16–44.

**Fig 9:**
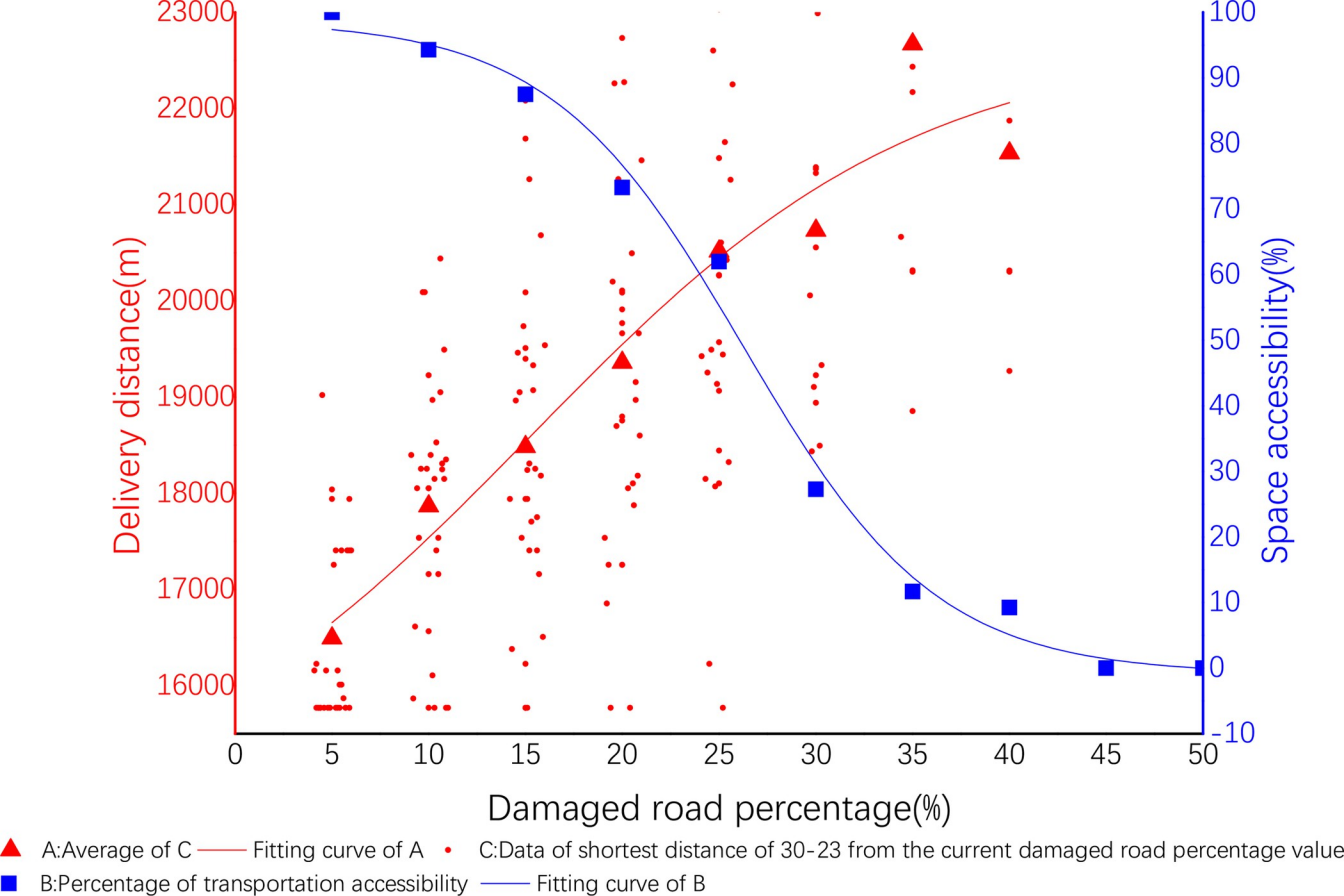
Delivery cost and spatial accessibility of 30–23.

**Fig 10:**
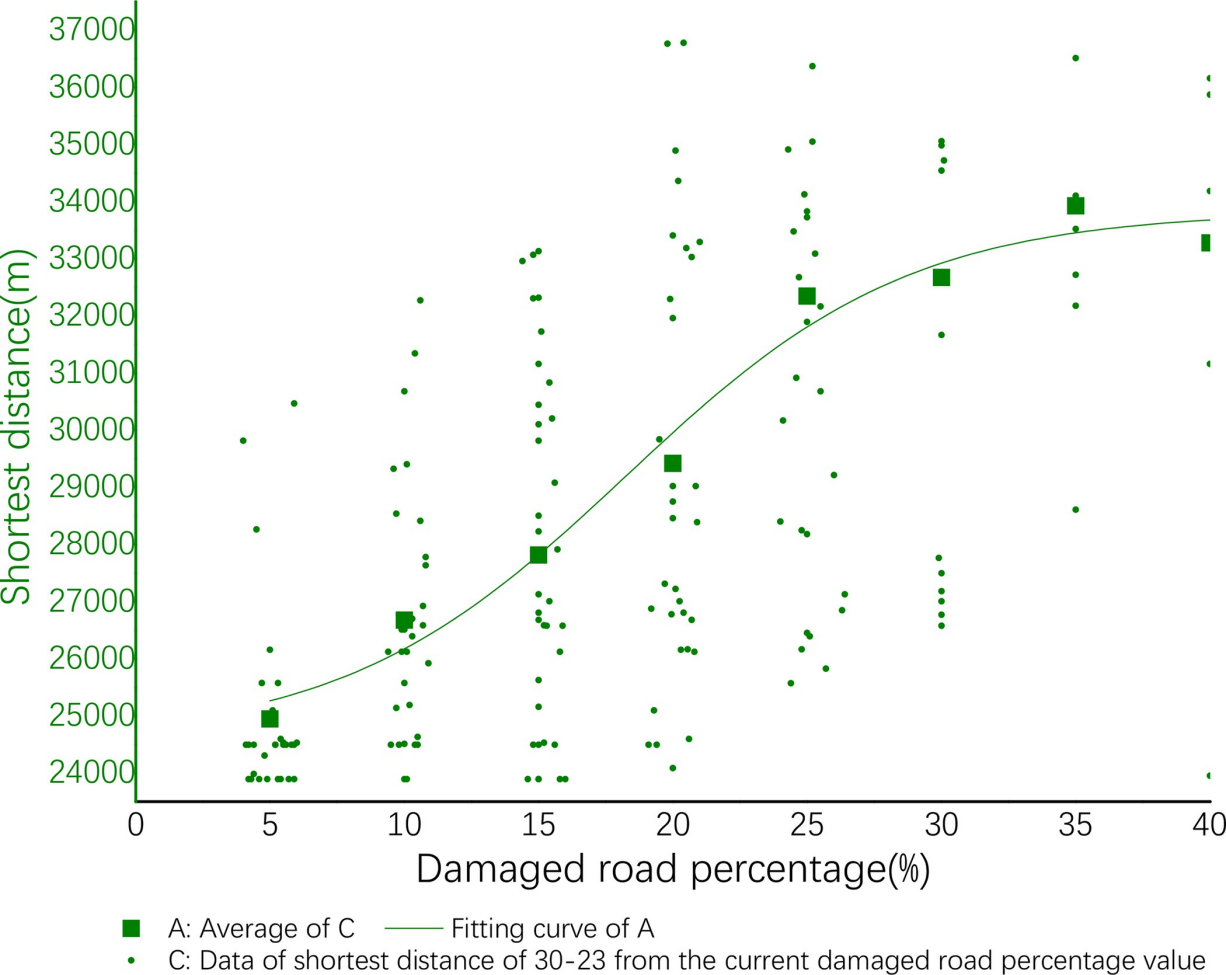
Delivery efficiency of 30–23.

The same regression analysis is performed according to the experiments above, then the following results are obtained. Test data as shown in Tables 17–22. The result graph in Figs 7–10. The analysis obtain similar regularity to above tests.

**Table 17:** Test data of distribution cost of 16–44.

Percentage of road damage (%)	Data of shortest distance of 16–44 from the current damaged road percentage value(m)	Average delivery distance (m)
5	13592,13152,13152,13152,13152,14923,13171,13152,13152,13152,13592,15770,13152,14390,13171,13171,14015,13152,14904,13152	13706
	13592,14904,14904,13152,13152,13592,13152,15770,13152,13592	
10	15090,15429,13152,17605,15073,15513,16023,16023,17060,13152,14904,19426,13171,13152,13152,13592,13611,15994,14455,15861	15117.4
	15139,15455,16741,15073,15073,15994,13152,15073,15880,14904,14904,14034,15601,17478,13171	
15	15975,15752,15221,23006,19934,15861,14234,14904,15092,17824,13171,15674,17731,13592,15582,14923,14390,15436,15073,14371	15531.9
	15861,16023,15975,13469,14904,17624,13152,16580,15752,13171,13592,13171	
20	15861,14904,14590,14637,25607,13171,17852,17615,16318,15994,21119,15719,13611,19751,15994,17874,14904,18924,14923,18798,20509,14637,17624,15601,14637,17844,15994,15092,15436,15955,19848	16817.5
25	18809,17090,17350,13611,15092,16871,16733,15164,16921,22305,15071,15532,16299,15030,16724,18982,15621,14904,18497,15455,20313,18408,17699,17670,18334,21201,23401,18675,15959,15752,21249,14904,16253,15655,18171,25518,18591,20765	17646.8
30	18147,17663,16468,17981,18511,16023,24611,17803,15513,25363,16786,19598,20233,19095,14904	18579.9
35	20176,15971,17411,18409,20662,15532,20164,21174,19280	18753.2
40	19194,16023,18135,21456	18702
45	None	None
50	None	None

**Table 18:** Simulation tests of spatial accessibility of 16–44.

**Percentage of road damage (%)**	5	10	15	20	25	30	35	40	45	50
**Number of experiment**	30	35	40	45	50	55	60	65	70	75
**Number of delivery availability**	30	35	32	31	38	15	9	4	0	0
**Accessibility (%)**	100	100	80	68.9	76	27.3	15	6.15	0	0

**Table 19:** Test data of distribution efficiency of 16–44.

Percentage of road damage (%)	Data of shortest distance of 16–44 from the current damaged road percentage value(m)	Average delivery distance (m)
5	22728,20963,20963,20066,20066,21273,20066,20066,20066,20066,22728,23132,20066,21273,20066,20066,21273,20066,21273,20066,21273,21273,21466,20066,20066,24033,20066,22170,20066,22728,	20984.6
10	24128,24943,20066,25525,22685,24029,23132,22752,25250,20066,21273,27023,20066,20066,20066,22728,22728,25656,24128,21816,23229,22752,23488,22685,22685,25656,20066,22685,21816,21273,21273,21273,23105,23945,20066	22689.2
15	26553,23105,22974,33254,34701,21816,22170,22170,20066,26425,20066,25832,26088,21273,23996,21273,22238,22752,22684,22238,20066,24029,25656,21767,21273,26105,20963,24006,23105,20066,22728,20066	23484.5
20	21816,21273,23135,22167,43139,20066,28377,25370,24719,25656,30637,24134,21273,34183,25656,24968,22578,32921,21273,27858	25686.3
	34524,21270,24800,23105,21270,26861,25371,22684,24057,24085,27050	
25	28186,24853,27072,22728,22685,26269,26045,26356,23956,33449,25433,23105,24152,25797,23838,28720,27423,22578,29485,22752,27746,32174,24968,26474,26881,29289,38820,29271,25899,24002,30341,22578,25462,23649,32455,44240,29399,27874	27273.8
30	33613,26808,23681,28819,31243,23132,35183,25923,26651,37460,25529,31465,28711,28047,22578	28598.5
35	32350,24002,27936,29186,32434,25347,29287,32731,26670	28882.6
40	27586,24437,32491,32982	29374
45	None	None
50	None	None

**Table 20:** Test data of distribution cost of 30–23.

Percentage of road damage (%)	Data of shortest distance of 16–44 from the current damaged road percentage value(m)	Average delivery distance (m)
5	18039,16156,15770,15770,15770,19021,15770,16156,15770,15770,17940,17255,15770,15770,15770,16010,15869,15770,17405,15770,17405,17405,17405,16156,16010,17405,15770,15770,16227,17940	16493.8
10	18397,15866,16613,18052,17536,18254,20090,20090,18254,17160,18397,16106,15770,18528,17536,20440,18309,19493,15770,15770,18350,18148,18247,19050,17160,17405,18148,18971,18052,15770,18052,19226,16566	17865.9
15	22081,18242,17405,17705,19071,18254,17405,17160,20681,16507,19538,18183,17750,19330,18309,17940,17536,19461,23079,17940,16381,18964,19050,19737,15770,21266,21686,16227,15770,19397,15770,19508,20088,17940,15770	18482.9
20	17536,16853,17255,15770,20199,22262,18699,21266,21214,22732,22273,19345,18052,25519,20495,17876,19156,18183,18600,21461,19663,18971,18103,15770,18756,18798,17255,20085,19663,17255,20106,19767,19912	19359.1
25	19425,18071,18148,19255,16227,19493,22603,20449,19137,23030,20606,15770,21650,20424,26016,21258,22250,18324,19441,21483,20363,25737,23420,18444,19065,18103,20270,19570,20262,23308,24408	20516.45
30	20056,18435,19105,21329,22989,18495,19332,20318,21373,24759,24691,21389,19226,18940,20555	20732.8
35	20665,25987,22433,24148,22170,18855,24427	22669.3
40	20303,23364,19272,21874,24096,20315	21537.3
45	None	None
50	None	None

**Table 21:** Simulation tests of spatial accessibility of 30–23.

**Percentage of road damage (%)**	5	10	15	20	25	30	35	40	45	50
**Number of experiment**	30	35	40	45	50	55	60	65	70	75
**Number of delivery availability**	30	33	35	33	31	15	7	6	0	0
**Accessibility (%)**	100	94.3	87.5	73.3	62	27.27	11.67	9.23	0	0

**Table 22:** Test data of distribution efficiency of 30–23.

Percentage of road damage (%)	Data of shortest distance of 16–44 from the current damaged road percentage value(m)	Average delivery distance (m)
5	29816,24495,23892,23892,23982,28263,23892,25577,24304,23892,26156,25096,24495,23892,23892,24529,24495,23892,24495,23892,24529,24495,24495,25577,24597,24495,24495,23892,24495,30470	24945.97
10	30681,23892,25577,26122,24495,29325,28540,24495,26511,26681,29404,25190,26394,31345,24495,32271,26587,27636,25923,24512	26674.4
	26511,27779,26923,28413,24631,24495,26701,26681,26122,23892,26122,26769,25140	
15	33133,31726,24529,26578,27004,30205,24495,27915,23892,26578,23892,26122,29080,30833,26587,27127,24495,23892,32960,29816,26681,23892,31162,30102,25628,33070,30446,25160,23892,28503,26806,28227,32308,32318,24495	27815.7
20	24495,26875,25096,24495,29838,38083,27314,36764,32296,33407,34896,34364,26156,36781,33187,24597,33030,26122,28391,33294,29022,26681,26163,26806,27004,27221,26776,28459,29021,29334,28750,31964,24085	29417.2
25	28402,30170,28247,34912,25572,33478,30916,32676,26163,34128,33727,26394,35050,33089,44085,32168,39060,25828,30681,36371,29215,41625,38850,26849,27127,26453,28183,33829,31894,39712,37964,	32348.97
30	27496,26578,34986,34720,39626,27181,27766,35060,34543,44508,39534,31666,26769,27004	32674.1
35	32720,39835,33522,34103,32180,28608,36512	33925.7
40	38339,36161,31162,35874,34186,23952	33279
45	None	None
50	None	None

#### 1) Delivery cost of 16–44

The coefficients of nonlinear fitting regression equation of cost are shown in Table 23 below. The nonlinear fitting regression equation corresponding to this coefficient shown by (3). Among them A_1_ = 12525.64691, A_2_ = 19110.29622, X_0_ = 15.13598, d_x_ = 7.45148. See (10) for details:

**Table 23:** Fitting equation parameters of cost of 16–44.

A_1_	A_2_	x_0_	d_x_
12525.64691	19110.29622	15.13598	7.45148


$$y=19110.29622+\left(-6584.64931/(1+exp\left(\left(x-x_{0}\right)15.13598/7.45148\right)\right)$$ (10)

#### 2) Spatial accessibility of 16–44

The spatial accessibility distribution is shown in Fig 7, and the spatial accessibility experimental parameters are shown in Table 18 below.

The coefficients of nonlinear fitting regression equation of accessibility are shown in Table 24 below. The nonlinear fitting regression equation corresponding to this coefficient shown by (5). Among them A_1_ = 98.06584, A_2_ = -3.14754, X_0_ = 27.42254, p = 5.29003. See (11) for details:

**Table 24:** Fitting equation parameters of accessibility of 16–44.

A_1_	A_2_	x_0_	p
98.06584	-3.14754	27.42254	5.29003


y=−3.14754+101.21338/(1+((x/27.42254)^5.29003) (11)

#### 3) Delivery efficiency of 16–44

The coefficients of nonlinear fitting regression equation of efficiency are shown in Table 25 below. The nonlinear fitting regression equation corresponding to this coefficient shown by (3). Among them A_1_ = 19996.45965, A_2_ = 29673.17443, X_0_ = 17.77651, d_x_ = 6.48962. See (12) for details:

**Table 25:** Fitting equation parameters of efficiency of 16–44.

A_1_	A_2_	x_0_	d_x_
19996.45965	29673.17443	17.77651	6.48962


y=29673.17443+(−9676.71478/(1+exp((x−17.77651)/6.48962)) (12)

#### 4) Delivery cost of 30–23

The coefficients of nonlinear fitting regression equation of cost are shown in Table 26 below. The nonlinear fitting regression equation corresponding to this coefficient shown by (3). Among them A_1_ = 14498.48429, A_2_ = 22697.81663, X_0_ = 15.28431, d_x_ = 9.99097. See (13) for details:

**Table 26:** Fitting equation parameters of cost of 30–23.

A_1_	A_2_	x_0_	d_x_
14498.48429	22697.81663	15.28431	9.99097


y=22697.81663+(−8199.33234)/(1+exp((x−15.28431)/9.99097) (13)

#### 5) Spatial accessibility of 30–23

The coefficients of nonlinear fitting regression equation of spatial accessibility are shown in Table 27 below. The nonlinear fitting regression equation corresponding to this coefficient shown by (5). Among them A_1_ = 98.74033, A_2_ = -0.85794, X_0_ = 26.2723, p = 4.98714. See (14) for details:

**Table 27:** Fitting equation parameters of accessibility of 30–23.

A_1_	A_2_	x_0_	p
98.74033	-0.85794	26.2723	4.98714


y=−0.85794+99.59827/(1+((x/26.2723)^4.98714) (14)

#### 6) Delivery efficiency of 30–23

The coefficients of nonlinear fitting regression equation of efficiency are shown in Table 28 below. The nonlinear fitting regression equation corresponding to this coefficient shown by (3). Among them A_1_ = 24564.32919, A_2_ = 33830.21604, X_0_ = 18.24959, d_x_ = 5.28775. See (15) for details:

**Table 28:** Fitting equation parameters of efficiency of 30–23.

A_1_	A_2_	x_0_	d_x_
24564.32919	33830.21604	18.24959	5.28775


y=33830.21604+(−9265.88685/(1+exp((x−18.24959)/5.28775)) (15)

The control tests identify that the results satisfy the obtained delivery curve by changing different delivery tasks. In these three indicators, the percentage of damaged roads to the control tests have the same values of the corners of the curve, which verifies the universality of the three results of distribution costs, distribution accessibility and distribution efficiency, and concludes that the road network has a general rule for delivery. These results offer new ideas about future research on the road network delivery.

## 5. Conclusions

This research is an extension of the static road network to solve the delivery problem. Due to the uncertainty of the occurrence time of large-scale natural calamities, the affected roads have varying degrees of damage and duration. Over time, the number and extent of damaged roads vary due to the impact of natural calamities. Therefore, the delivery of emergency supplies or evacuation routes are dynamically changing.

This study shows:

1. The results obtained through a large number of experiments show that when an emergency occurs, the distribution of emergency materials distribution cost, space accessibility and distribution efficiency under the percentage of road damage has a certain regularity, and the fitting curve presents obvious S-curve changes. There is an obvious inflection point in these experiment when the damaged road percentage reaches a certain value, with 15%-20% and 30%-35%.
2. This method can be used to maximize time efficiency and reduce the losses of disaster according to the most suitable delivery path and delivery time. It can support emergency commanders to make better decisions, improve the delivery efficiency of emergency supplies, and transfer or evacuate citizens.
3. The number of damaged and impassable roads under different conditions will affect all aspects of the allocation, such as distribution costs, spatial accessibility and distribution efficiency of the road network.

Future researches can increase the number of simulations to obtain more obvious rule patterns. Meanwhile, more researches are need to be developed on time window constrains, road environmental factors and other dynamic scenarios of agent’s dynamic delivery simulation problems.

## Supporting information

S1 Appendix (DOCX)
