# Supplementary material for: A two-stage simulation analysis of uncertain road damage on the urban emergency delivery network
Source: PLoS One. 2022 May 25;17(5):e0267043. doi: 10.1371/journal.pone.0267043 (PMC9132276; doi:10.1371/journal.pone.0267043)
Supplement: S1 Appendix — (DOCX) [file pone.0267043.s001.docx]

S1 Appendix

Appendix A. Attributes of links of the road network

| Attribute | Road number | Start node number | End node number | Distance(m) | Speed level  (km/h) |
| --- | --- | --- | --- | --- | --- |
| Value | 1 | 18 | 65 | 527 | 60-80 |
|  | 2 | 65 | 6 | 536 | 60-80 |
|  | 3 | 44 | 6 | 1504 | 60-80 |
|  | 4 | 6 | 36 | 1489 | 40-60 |
|  | 5 | 68 | 44 | 666 | 40-60 |
|  | 6 | 44 | 50 | 290 | 60-80 |
|  | 7 | 68 | 50 | 576 | 0-30 |
|  | 8 | 50 | 36 | 866 | 0-30 |
|  | 9 | 36 | 68 | 807 | 0-30 |
|  | 10 | 66 | 68 | 1275 | 40-60 |
|  | 11 | 66 | 60 | 1208 | 30-40 |
|  | 12 | 71 | 39 | 1285 | 0-30 |
|  | 13 | 40 | 71 | 1254 | 40-60 |
|  | 14 | 36 | 66 | 766 | 30-40 |
|  | 15 | 39 | 60 | 678 | 40-60 |
|  | 16 | 39 | 40 | 647 | 30-40 |
|  | 17 | 53 | 39 | 791 | 30-40 |
|  | 18 | 53 | 42 | 1368 | 60-80 |
|  | 19 | 53 | 12 | 2813 | 60-80 |
|  | 20 | 42 | 33 | 1067 | 40-60 |
|  | 21 | 33 | 46 | 1409 | 30-40 |
|  | 22 | 46 | 42 | 991 | 40-60 |
|  | 23 | 33 | 80 | 636 | 40-60 |
|  | 24 | 12 | 58 | 1421 | 30-40 |
|  | 25 | 58 | 79 | 853 | 0-30 |
|  | 26 | 58 | 74 | 1449 | 0-30 |
|  | 27 | 79 | 78 | 2358 | 0-30 |
|  | 28 | 25 | 74 | 547 | 30-40 |
|  | 29 | 78 | 25 | 313 | 30-40 |
|  | 30 | 78 | 64 | 965 | 40-60 |
|  | 31 | 64 | 76 | 1106 | 0-30 |
|  | 32 | 29 | 30 | 1373 | 60-80 |
|  | 33 | 30 | 59 | 1072 | 40-60 |
|  | 34 | 29 | 59 | 687 | 0-30 |
|  | 35 | 59 | 73 | 1731 | 40-60 |
|  | 36 | 76 | 73 | 865 | 30-40 |
|  | 37 | 47 | 64 | 2444 | 0-30 |
|  | 38 | 73 | 31 | 1286 | 30-40 |
|  | 39 | 31 | 47 | 234 | 0-30 |
|  | 40 | 73 | 24 | 1507 | 30-40 |
|  | 41 | 24 | 56 | 722 | 0-30 |
|  | 42 | 56 | 16 | 1018 | 60-80 |
|  | 43 | 24 | 16 | 521 | 40-60 |
|  | 44 | 16 | 8 | 2337 | 60-80 |
|  | 45 | 2 | 45 | 3424 | 30-40 |
|  | 46 | 26 | 47 | 1381 | 0-30 |
|  | 47 | 74 | 26 | 1335 | 40-60 |
|  | 48 | 26 | 2 | 544 | 60-80 |
|  | 49 | 52 | 2 | 341 | 0-30 |
|  | 50 | 52 | 43 | 1005 | 0-30 |
|  | 51 | 43 | 46 | 904 | 30-40 |
|  | 52 | 57 | 52 | 1736 | 40-60 |
|  | 53 | 46 | 75 | 1419 | 0-30 |
|  | 54 | 42 | 75 | 1465 | 30-40 |
|  | 55 | 75 | 57 | 1213 | 30-40 |
|  | 56 | 75 | 41 | 1471 | 40-60 |
|  | 57 | 10 | 40 | 1638 | 0-30 |
|  | 58 | 41 | 10 | 1051 | 30-40 |
|  | 59 | 41 | 48 | 1305 | 0-30 |
|  | 60 | 34 | 10 | 1390 | 0-30 |
|  | 61 | 10 | 72 | 1285 | 40-60 |
|  | 62 | 48 | 72 | 1546 | 30-40 |
|  | 63 | 48 | 57 | 2116 | 30-40 |
|  | 64 | 57 | 22 | 1900 | 60-80 |
|  | 65 | 22 | 13 | 1214 | 30-40 |
|  | 66 | 13 | 45 | 2216 | 30-40 |
|  | 67 | 45 | 32 | 1875 | 60-80 |
|  | 68 | 8 | 28 | 1618 | 40-60 |
|  | 69 | 8 | 54 | 1450 | 30-40 |
|  | 70 | 54 | 38 | 695 | 60-80 |
|  | 71 | 38 | 9 | 654 | 40-60 |
|  | 72 | 28 | 54 | 450 | 0-30 |
|  | 73 | 9 | 28 | 303 | 30-40 |
|  | 74 | 28 | 32 | 800 | 60-80 |
|  | 75 | 32 | 63 | 299 | 0-30 |
|  | 76 | 63 | 21 | 1454 | 0-30 |
|  | 77 | 63 | 13 | 1643 | 30-40 |
|  | 78 | 13 | 21 | 1361 | 30-40 |
|  | 79 | 21 | 1 | 1474 | 0-30 |
|  | 80 | 21 | 4 | 1116 | 60-80 |
|  | 81 | 1 | 22 | 1193 | 60-80 |
|  | 82 | 1 | 4 | 502 | 30-40 |
|  | 83 | 4 | 69 | 275 | 30-40 |
|  | 84 | 69 | 7 | 501 | 0-30 |
|  | 85 | 7 | 62 | 256 | 30-40 |
|  | 86 | 5 | 62 | 1411 | 30-40 |
|  | 87 | 69 | 3 | 1909 | 30-40 |
|  | 88 | 22 | 3 | 746 | 60-80 |
|  | 89 | 3 | 35 | 1007 | 30-40 |
|  | 90 | 3 | 5 | 1026 | 40-60 |
|  | 91 | 5 | 35 | 938 | 60-80 |
|  | 92 | 35 | 67 | 1665 | 60-80 |
|  | 93 | 67 | 5 | 1175 | 30-40 |
|  | 94 | 70 | 67 | 279 | 0-30 |
|  | 95 | 61 | 70 | 1028 | 30-40 |
|  | 96 | 70 | 11 | 1645 | 60-80 |
|  | 97 | 11 | 23 | 1599 | 30-40 |
|  | 98 | 15 | 11 | 2941 | 40-60 |
|  | 99 | 23 | 37 | 419 | 0-30 |
|  | 100 | 23 | 27 | 449 | 30-40 |
|  | 101 | 17 | 27 | 221 | 30-40 |
|  | 102 | 27 | 77 | 1505 | 30-40 |
|  | 103 | 55 | 17 | 446 | 30-40 |
|  | 104 | 77 | 55 | 1078 | 0-30 |
|  | 105 | 20 | 77 | 884 | 60-80 |
|  | 106 | 77 | 19 | 1045 | 60-80 |
|  | 107 | 20 | 19 | 426 | 30-40 |
|  | 108 | 15 | 20 | 408 | 30-40 |
|  | 109 | 34 | 20 | 423 | 0-30 |
|  | 110 | 19 | 34 | 361 | 30-40 |
|  | 111 | 72 | 34 | 327 | 30-40 |
|  | 112 | 60 | 53 | 436 | 0-30 |
|  | 113 | 80 | 12 | 305 | 30-40 |
|  | 114 | 71 | 66 | 431 | 40-60 |
|  | 115 | 14 | 49 | 430 | 30-40 |
|  | 116 | 61 | 14 | 422 | 40-60 |
|  | 117 | 62 | 14 | 520 | 40-60 |
|  | 118 | 49 | 61 | 331 | 30-40 |
|  | 119 | 62 | 49 | 219 | 0-30 |
|  | 120 | 41 | 40 | 860 | 40-60 |
|  | 121 | 45 | 8 | 468 | 30-40 |
|  | 122 | 65 | 44 | 1782 | 0-30 |
|  | 123 | 6 | 60 | 2295 | 60-80 |
|  | 124 | 79 | 30 | 2566 | 40-60 |
|  | 125 | 59 | 56 | 2497 | 60-80 |
|  | 126 | 71 | 19 | 3104 | 40-60 |
|  | 127 | 35 | 48 | 1144 | 60-80 |
|  | 128 | 74 | 43 | 499 | 30-40 |
|  | 129 | 76 | 29 | 434 | 30-40 |
|  | 130 | 72 | 15 | 216 | 60-80 |

Appendix B. Data of road network

| Road number | Road grades | D_0_ | D_n_ |
| --- | --- | --- | --- |
| 1 | 1 | 527 | 527 |
| 2 | 1 | 536 | 536 |
| 3 | 1 | 1504 | 1504 |
| 4 | 2 | 1489 | 1861 |
| 5 | 2 | 666 | 833 |
| 6 | 1 | 290 | 290 |
| 7 | 4 | 576 | 1440 |
| 8 | 4 | 866 | 2165 |
| 9 | 4 | 807 | 2018 |
| 10 | 2 | 1275 | 1594 |
| 11 | 3 | 1208 | 2013 |
| 12 | 4 | 1285 | 3213 |
| 13 | 2 | 1254 | 1568 |
| 14 | 3 | 766 | 1277 |
| 15 | 2 | 678 | 848 |
| 16 | 3 | 647 | 1078 |
| 17 | 3 | 791 | 1318 |
| 18 | 1 | 1368 | 1368 |
| 19 | 1 | 2813 | 2813 |
| 20 | 2 | 1067 | 1334 |
| 21 | 3 | 1409 | 2348 |
| 22 | 2 | 991 | 1239 |
| 23 | 2 | 636 | 795 |
| 24 | 3 | 1421 | 2368 |
| 25 | 4 | 853 | 2133 |
| 26 | 4 | 1449 | 3623 |
| 27 | 4 | 2358 | 5895 |
| 28 | 3 | 547 | 912 |
| 29 | 3 | 313 | 522 |
| 30 | 2 | 965 | 1206 |
| 31 | 4 | 1106 | 2765 |
| 32 | 1 | 1373 | 1373 |
| 33 | 2 | 1072 | 1340 |
| 34 | 4 | 687 | 1718 |
| 35 | 2 | 1731 | 2164 |
| 36 | 3 | 865 | 1442 |
| 37 | 4 | 2444 | 6110 |
| 38 | 3 | 1286 | 2143 |
| 39 | 4 | 234 | 585 |
| 40 | 3 | 1507 | 2512 |
| 41 | 4 | 722 | 1805 |
| 42 | 1 | 1018 | 1018 |
| 43 | 2 | 521 | 651 |
| 44 | 1 | 2337 | 2337 |
| 45 | 3 | 3424 | 5707 |
| 46 | 4 | 1381 | 3453 |
| 47 | 2 | 1335 | 1669 |
| 48 | 1 | 544 | 544 |
| 49 | 4 | 341 | 853 |
| 50 | 4 | 1005 | 2513 |
| 51 | 3 | 904 | 1507 |
| 52 | 2 | 1736 | 2170 |
| 53 | 4 | 1419 | 3548 |
| 54 | 3 | 1465 | 2442 |
| 55 | 3 | 1213 | 2022 |
| 56 | 2 | 1471 | 1839 |
| 57 | 4 | 1638 | 4095 |
| 58 | 3 | 1051 | 1752 |
| 59 | 4 | 1305 | 3263 |
| 60 | 4 | 1390 | 3475 |
| 61 | 2 | 1285 | 1606 |
| 62 | 3 | 1546 | 2577 |
| 63 | 3 | 2116 | 3527 |
| 64 | 1 | 1900 | 1900 |
| 65 | 3 | 1214 | 2023 |
| 66 | 3 | 2216 | 3693 |
| 67 | 1 | 1875 | 1875 |
| 68 | 2 | 1618 | 2023 |
| 69 | 3 | 1450 | 2417 |
| 70 | 1 | 695 | 695 |
| 71 | 2 | 654 | 818 |
| 72 | 4 | 450 | 1125 |
| 73 | 3 | 303 | 505 |
| 74 | 1 | 800 | 800 |
| 75 | 4 | 299 | 748 |
| 76 | 4 | 1454 | 3635 |
| 77 | 3 | 1643 | 2738 |
| 78 | 3 | 1361 | 2268 |
| 79 | 4 | 1474 | 3685 |
| 80 | 1 | 1116 | 1116 |
| 81 | 1 | 1193 | 1193 |
| 82 | 3 | 502 | 837 |
| 83 | 3 | 275 | 458 |
| 84 | 4 | 501 | 1253 |
| 85 | 3 | 256 | 427 |
| 86 | 3 | 1411 | 2352 |
| 87 | 3 | 1909 | 3182 |
| 88 | 1 | 746 | 746 |
| 89 | 3 | 1007 | 1678 |
| 90 | 2 | 1026 | 1283 |
| 91 | 1 | 938 | 938 |
| 92 | 1 | 1665 | 1665 |
| 93 | 3 | 1175 | 1958 |
| 94 | 4 | 279 | 698 |
| 95 | 3 | 1028 | 1713 |
| 96 | 1 | 1645 | 1645 |
| 97 | 3 | 1599 | 2665 |
| 98 | 2 | 2941 | 3676 |
| 99 | 4 | 419 | 1048 |
| 100 | 3 | 449 | 748 |
| 101 | 3 | 221 | 368 |
| 102 | 3 | 1505 | 2508 |
| 103 | 3 | 446 | 743 |
| 104 | 4 | 1078 | 2695 |
| 105 | 1 | 884 | 884 |
| 106 | 1 | 1045 | 1045 |
| 107 | 3 | 426 | 710 |
| 108 | 3 | 408 | 680 |
| 109 | 4 | 423 | 1058 |
| 110 | 3 | 361 | 602 |
| 111 | 3 | 327 | 545 |
| 112 | 4 | 436 | 1090 |
| 113 | 3 | 305 | 508 |
| 114 | 2 | 431 | 539 |
| 115 | 3 | 430 | 717 |
| 116 | 2 | 422 | 528 |
| 117 | 2 | 520 | 1300 |
| 118 | 3 | 331 | 552 |
| 119 | 4 | 219 | 548 |
| 120 | 2 | 860 | 1075 |
| 121 | 3 | 468 | 780 |
| 122 | 4 | 1782 | 4455 |
| 123 | 1 | 2295 | 2295 |
| 124 | 2 | 2566 | 3208 |
| 125 | 1 | 2497 | 2497 |
| 126 | 2 | 3104 | 3880 |
| 127 | 1 | 1144 | 1144 |
| 128 | 3 | 499 | 832 |
| 129 | 3 | 434 | 723 |
| 130 | 1 | 216 | 216 |
